# Untangling the fungal niche: the trait-based approach

**DOI:** 10.3389/fmicb.2014.00579

**Published:** 2014-10-31

**Authors:** Thomas W. Crowther, Daniel S. Maynard, Terence R. Crowther, Jordan Peccia, Jeffrey R. Smith, Mark A. Bradford

**Affiliations:** ^1^Yale School of Forestry and Environmental Studies, Yale UniversityNew Haven, CT, USA; ^2^Yale UniversityCT, USA (retired); ^3^Department of Chemical and Environmental Engineering, Yale UniversityNew Haven, CT, USA

**Keywords:** fungal niche, functional traits, fungal biogeogoraphy, community ecology, dominance-tolerance trade-off

## Abstract

Fungi are prominent components of most terrestrial ecosystems, both in terms of biomass and ecosystem functioning, but the hyper-diverse nature of most communities has obscured the search for unifying principles governing community organization. In particular, unlike plants and animals, observational studies provide little evidence for the existence of niche processes in structuring fungal communities at broad spatial scales. This limits our capacity to predict how communities, and their functioning, vary across landscapes. We outline how a shift in focus, from taxonomy toward functional traits, might prove to be valuable in the search for general patterns in fungal ecology. We build on theoretical advances in plant and animal ecology to provide an empirical framework for a trait-based approach in fungal community ecology. Drawing upon specific characteristics of the fungal system, we highlight the significance of drought stress and combat in structuring free-living fungal communities. We propose a conceptual model to formalize how trade-offs between stress-tolerance and combative dominance are likely to organize communities across environmental gradients. Given that the survival of a fungus in a given environment is contingent on its ability to tolerate antagonistic competitors, measuring variation in combat trait expression along environmental gradients provides a means of elucidating realized, from fundamental niche spaces. We conclude that, using a trait-based understanding of how niche processes structure fungal communities across time and space, we can ultimately link communities with ecosystem functioning. Our trait-based framework highlights fundamental uncertainties that require testing in the fungal system, given their potential to uncover general mechanisms in fungal ecology.

## Introduction

Microorganisms are important regulators of global biogeochemical cycling, responsible for mineralising organic nutrients and governing the exchanges of carbon and nutrients between the biosphere and atmosphere. As such, the inclusion of microbial processes into Earth system models (ESM) is integral to our understanding of biogeochemical cycles and their feedbacks to global climate change. Current models incorporate broad climate variables and plant traits as the dominant controls on microbial processes, due to their predictable distributions at global and regional scales (Bonan et al., 2013; Todd-Brown et al., 2013; Wieder et al., 2013; Bradford et al., 2014). A vast body of evidence, however, suggests that microbial-mediated ecosystem functioning can vary substantially within environments, depending on the community composition (Schimel and Schaeffer, 2012; Strickland et al., 2014; van der Wal et al., 2014). This variation in community performance has led researchers to question the validity of broad-scale indicators, highlighting the potential value of including local-scale controls such as direct microbial community attributes to explain and project biogeochemical cycling at global and regional scales (Bradford et al., 2014). Our capacity to incorporate such local community information is, however, restricted by a limited understanding of microbial biogeography or the patterns of microbial traits across broad spatial scales.

Untangling the mechanisms governing patterns in community structure across time and space are foundational goals of community ecology. Despite concerns that the complex nature of natural communities precludes the formulation of unifying principles (Simberloff, 2004), the Hutchinsonian niche concept remains a powerful framework to understand community patterns (Hutchinson, 1957). Like plant and animal communities, the importance of niche processes in shaping bacterial communities across landscapes is increasingly apparent (Barberán et al., 2014). In contrast, environmental characteristics consistently fail to explain the majority of the variation in fungal communities at broad spatial-scales, leading to the widespread belief that neutral processes (e.g., dispersal limitation) are dominant forces governing patterns in fungal taxa (Martiny et al., 2006; Feinstein and Blackwood, 2013; Wu et al., 2013; Cline and Zak, 2014; Talbot et al., 2014). However, the focus on taxonomy, rather than traits, is likely to have restricted the search for predictable patterns in community organization (Aguilar-Trigueros et al., 2014); because niche processes filter communities based on the expression of individual-level traits, it is likely that environmental characteristics are linked more closely to trait values than to taxonomy at broad spatial-scales.

The niche concept makes competitive advantage or persistence contingent on environmental conditions, given trade-offs in species traits that favor coexistence (Hutchinson, 1957). Multiple biotic and abiotic processes then serve as environmental filters, selecting for traits that allow individuals to survive and compete under those specific conditions. To understand niche processes, then, plant ecologists have invested tremendous efforts in studying communities through the lens of functional traits (Adler et al., 2013; Herben and Goldberg, 2014). By considering trade-offs in ecophysiological, morphological and life-history traits, this approach provides mechanistic linkages between fundamental biology, community dynamics and ecosystem functioning. Despite the prevalence of trait-based approaches in aboveground ecology, fungal ecologists traditionally view communities through a taxonomic lens, often resulting in the loss of ecological generality (Green et al., 2008; Allison, 2012; Powell et al., 2013). In 2013, for example, less than 2% of experimental studies published in the journal *Fungal Ecology* used traits or trade-offs to explain species patterns. In contrast, ~76% of the experimental studies published in the same year in the corresponding plant journal (*Journal of Ecology*) examined processes through the lens of functional traits. It is important to note that the expression of many traits is linked inexorably to taxonomic identity, so trait filtering often influences taxonomic compositions. However, statements about traits provide generality and predictability, whereas nomenclatural ecology tends toward highly-contingent rules and special cases (only providing information about the study species and closely related taxa; McGill et al., 2006; Adler et al., 2013). Analogous to the example from the plant-based literature (McGill et al., 2006), the statement “faster-growing, cord-forming fungi are more combative than litter-dwelling microfungi” is more useful than “*Resinicium bicolor* out-competes *Mortierella verticillata* in soil” (*sensu* Crowther et al., 2013).

In a classic trait-based framework, Grime (1977) characterized plants, animals and fungi as competitors (C), stress tolerators (S) or colonizers (i.e., ruderals; R), a set of distinctions that encompass the physiological traits governing species distributions and their influences on ecosystem processes. Theory in plant and animal ecology has built on this categorical framework to embrace continuous variation in the expression of various traits across multiple species. Despite calls for a focus on species- and individual-level variation in multiple functional traits in the mycorrhizal literature (Behm and Kiers, 2014; Koide et al., 2014; Parrent et al., 2010) often with a view to understanding host plant dynamics, the trait-based framework is yet to be extended throughout fungal ecology (Aguilar-Trigueros et al., 2014).

We discuss trade-offs in stress-tolerance and combative dominance to exemplify how a trait-based framework might aid in the search for general mechanisms within fungal community ecology. Drawing on examples from across the fungal kingdom, we focus on free-living groups because their physiology is directly linked to the external environment rather than the characteristics of host species. The manuscript is divided into three main sections. First, we discuss the need for a trait-based framework in fungal ecology. The second section proposes this framework, acknowledging that there is a paucity of continuous trait-based datasets for free-living fungi that precludes the empirical testing of specific trade-offs. Instead, we develop a conceptual framework, incorporating specific characteristics of the fungal system, which outlines how trade-offs between competitive ability and drought tolerance can improve our understanding of the processes structuring fungal communities. We believe this framework demonstrates the need to explore fungal traits within the context of environmental gradients and biotic interactions to understand the ecology of all fungal communities (Saunders et al., 2010; Aguilar-Trigueros et al., 2014). In the third section, we highlight the implications of our framework for understanding patterns in fungal communities across time and space. We conclude that trait-based approaches can provide a tractable set of tools for understanding the links between fungal community ecology and ecosystem ecology. These tools can facilitate the conversion of fungal ecology from a phenomenological discipline into a more mechanistic one.

## The basis for a trait-based approach

### The taxonomic approach in fungal ecology

The fungal kingdom encompasses a huge diversity of species, with micro- and macroscopic taxa that inhabit almost every habitat on Earth. Despite their global distribution, most fungi are generally inconspicuous due to their small size and cryptic lifestyles, living on organic matter in soil and water, or in association with other organisms. Given their inconspicuousness and the fact that many species are indistinguishable from one another without the use of molecular tools, it is not surprising that only 2–5% of the estimated 1.5–6 million fungal species have been formally classified (Blackwell, 2011), and there are few, if any, fungi for which the full geographic distribution is documented.

The practical limitations in identifying fungi and “binning” them into taxonomic groups restricts our capacity to extrapolate to other (even closely-related) species, and the challenges in determining species distributions across landscapes precludes the scaling-up of processes observed in individuals (Green et al., 2008). Furthermore, most fungi are highly plastic, with individuals displaying substantial spatial and temporal variation in morphology and physiology that can obscure differences in life-history strategy observed between taxa (Aguilar-Trigueros et al., 2014). Indeed, individual fungi can display competitive, ruderal or stress-tolerant morphotypes at different stages of development (Pugh and Boddy, 1988). A focus on species identities can, therefore, obscure the detection of niche patterns, depending on the timing of experimental observation. Given that trait expression relates to the strategies of individuals at any one point in time, and under a given set of circumstances, greater emphasis on trait-based approaches in fungal ecology is pragmatic both theoretically and empirically.

### Fungal functional traits

Proponents of the functional approach in fungal ecology have traditionally divided taxa into functional groups. These groups are based on trophic status (mycorrhizal, saprotrophic, pathogenic), and within these, based on size (microfungi and macrofungi), pigmentation (dark-pigmented and non-pigmented), morphology (e.g., phalanx and guerrilla), physiology (e.g., brown rot and white rot) and life-history strategies (e.g., endo-, ecto- and ericoid-mycorrhiza; Aguilar-Trigueros et al., 2014). Such categorizations provide useful guides to navigate the complex fungal kingdom; they allow for the exploration of within-group generalizations, between-group comparisons and they confirm the positive effect of functional diversity on ecosystem processes (Hättenschwiler et al., 2005; Crowther et al., 2013). Categorical groupings are, however, often limited in their capacity to isolate mechanisms because species within the same groups can display a wide range of trait values (Naeem and Wright, 2003). Many plant endophytes can, for example, be considered early decomposers, as they initiate fungal succession in dead wood (Boddy, 2001), and there is a continuum of life-history strategies between purely pathogenic, mycorrhizal and saprotrophic fungi (Aguilar-Trigueros et al., 2014).

Following aboveground community ecology (e.g., McGill et al., 2006), we refer to “functional traits” as measurable properties of organisms that are used comparatively across individuals and influence an organism's performance or fitness. Measurement of traits on a continuous scale permits regression designs that maximize the predictive power for species responses or effects. Such scales of trait expression can inform community ecologists about the relative distributions of species along environmental filters (Webb et al., 2010; Lennon et al., 2012), and ecosystem ecologists about the specific components of a community that drive ecosystem functioning (Dias et al., 2013).

**Figure 1:**
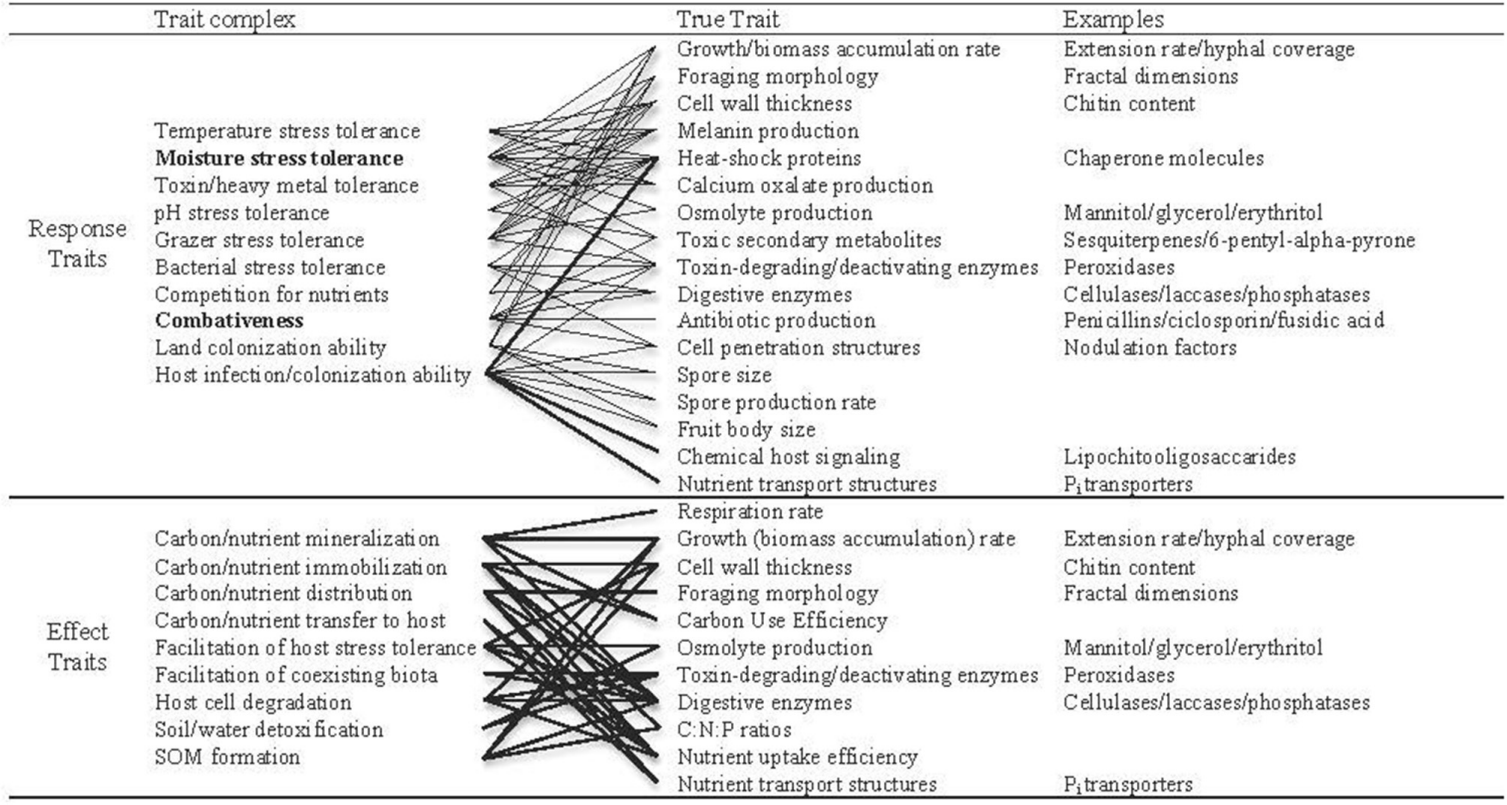
**Examples of trait complexes and corresponding true traits (lines connect trait complexes with the true traits that influence them), each of which can be measured in terms of constitutive or potential expression, and which exist on a continuum across saprotrophic, mycorrhizal and pathogenic fungi**. Even the true traits might be considered trait complexes as they are encoded for by complex sets of genes. Nevertheless, these are all measurable properties individuals that potentially relate to survival under a given set of environmental conditions. These traits can be measured directly or indirectly (by measuring functional gene expression), but are most useful when expression can be related to individuals. Response traits are those that dictate how organisms respond to biotic and abiotic conditions, and effect traits relate to an individual's effect on the environment. Several true traits are predicted to influence both response- and effect-trait complexes (Koide et al., 2014), and each trait complex could be comprised of a suite of interacting true traits. Highlighted in bold are the trait complexes that we propose are dominant structuring forces in free-living fungal communities.

In this paper, we refer to two categories of continuous traits: “trait complexes” and “true traits.” Trait complexes correspond to the performance of an individual relative to co-occurring taxa. These can relate to biotic (e.g., competitive ability) or abiotic factors (e.g., drought tolerance), and are a product of the expression of multiple true traits (e.g., growth rate or osmolyte production; Figure 1). Both categories can refer to response traits (that govern how organisms respond to different conditions) and effect traits (that determine how organisms affect their environment).

### Isolating and measuring fungal traits

Various techniques allow the measurement of fungal functional traits directly (by measuring expression) or indirectly (using transcriptomics to measure functional genes) across a range of habitats and hosts. In most cases, these traits can be measured at the community-level (Sinsabaugh et al., 2008) to provide a functional fingerprint of the community as a whole. This approach has benefitted from accelerating advances in sequencing-based (e.g., metatranscriptomic) techniques, which allow the mapping of functional genes or genome characteristics across ecosystems (Green et al., 2008; Barberán et al., 2014). A mechanistic understanding of functional trait organization within communities, however, requires the linking of trait expression with individuals (McGill et al., 2006). For example, having observed high levels of stress-tolerance traits in a given environment it is impossible to determine whether those traits are expressed by one or multiple individuals, and whether trait expression is a prerequisite for survival in that environment or not. The combined use of community-weighted trait measurements (direct or indirect) and metagenomic sequencing data is a powerful and widespread means of linking fungal trait expression with communities (Rajala et al., 2011; Crowther et al., 2013, 2014; Cline and Zak, 2014), but observed relationships between species and trait expression are purely correlative and provide no inferences about the organization of traits within the community. As with plant and animal ecology, a trait-based approach to understanding the structuring of fungal communities requires that trait, or functional gene, expression be attributed directly to individuals within the community. Currently, this generally requires the isolation of fungi.

The plastic nature of most fungal traits reflects that recorded in plants. Development and trait expression can vary drastically when grown under different conditions and on different substrates. As with plant ecology, fungal traits should therefore be estimated under optimal conditions, unless specific goals suggest otherwise (Peìrez-Harguindeguy et al., 2013). This allows the universal comparison of individual-level attributes, and the identification of fundamental physiological trade-offs. These patterns can then be used to understand community dynamics under different conditions (e.g., under different levels of stress or on substrates), where communities may be comprised of different taxa, but still governed by the same fundamental selection pressures.

## A framework for a trait-based approach

McGill et al. (2006) posit that a trait-based approach within a community context requires consideration of environmental gradients (habitat filters) and the interaction milieu (biotic interactions) to allow the selection of appropriate performance currencies (traits that reflect the performance of an individual in a given environment). In this section, we focus on a dominant environmental gradient (i.e., moisture availability) and biotic interaction (i.e., competition) to guide the selection of performance currencies for the fungal system. From this consideration, we build a framework for application of a trait-based approach in fungal ecology that relies on (i) identifying the dominant trade-offs (between trait complexes) that govern species distributions and abundances (Kneitel and Chase, 2004); and (ii) linking trait complex values with true traits to elucidate the mechanistic basis of these trade-offs and extrapolation beyond the study taxa (Lennon et al., 2012).

### Environmental gradients

**Figure 2:**
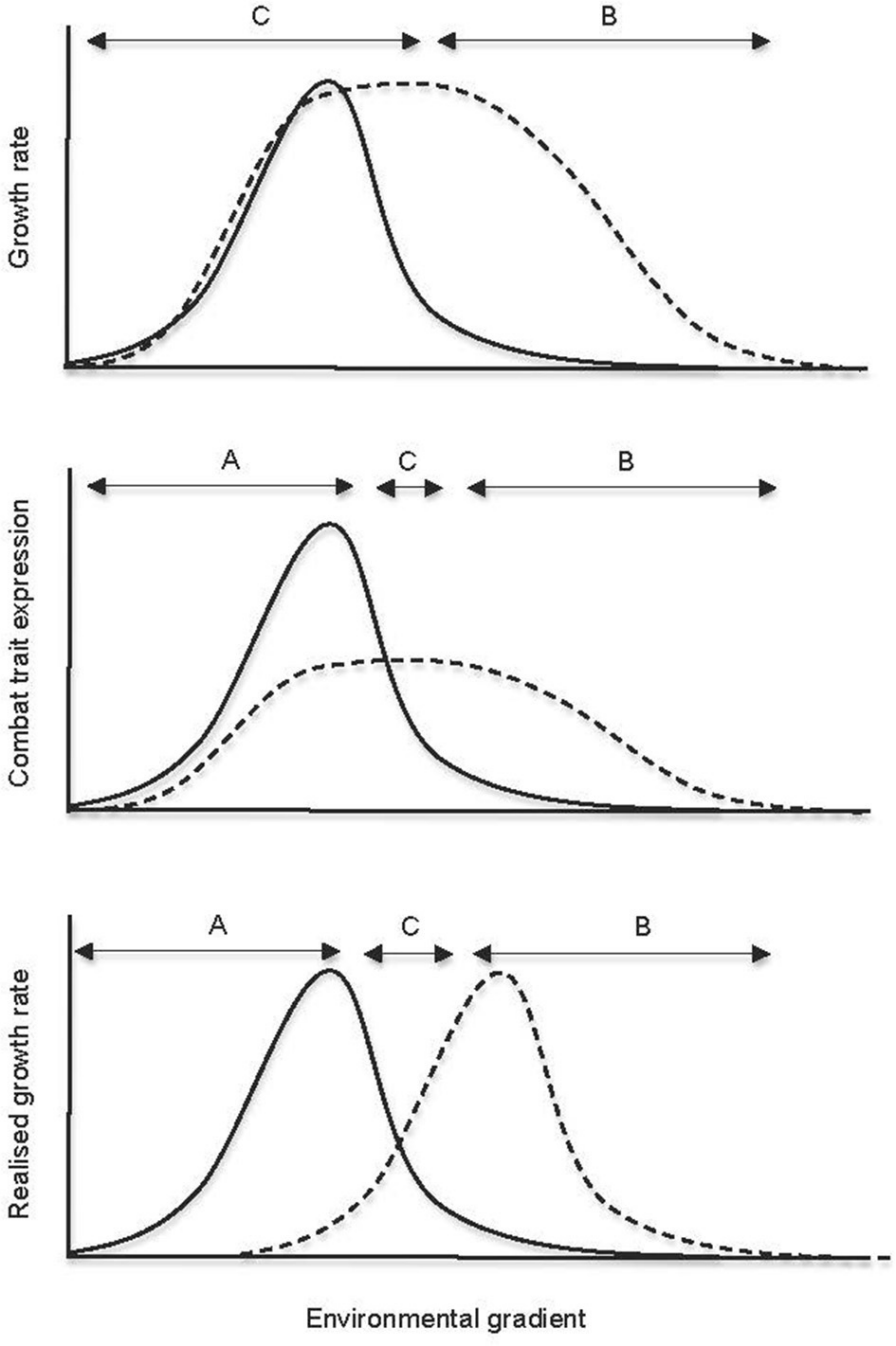
**Plotting the realized niche by estimating variation in a performance currency that represents the interaction milieu along an environmental gradient**. Traditional approaches to exploring individual niche spaces involve estimating survival (e.g., growth or respiration) across environmental gradients (fundamental niche), and then subsequently exploring biotic interactions within this space to approximate the realized niche. Our conceptual diagram shows how the measurement of traits associated with a dominant biotic process along the same environmental gradient can incorporate both biotic and abiotic filters and provide a meaningful estimation of an organism's realized niche. Niche differentiation between two species with identical growth rates can be visualized using this approach. The letters “A” and “B” refer to the dominance of those respective individuals at the corresponding point along the gradient, and “C” indicates coexistence. We stress that, even if maximum growth rates of the two fungi differed substantially from one another in plate “a,” the denoted niche spaces would remain unchanged, because growth in the absence of competitors does not imply growth during combat.

Identifying how survivorship varies along environmental gradients is the initial step in defining a species' fundamental niche, and this alone can provide a powerful tool to predict the structuring of communities at the broadest spatial scales (Green et al., 2008). As with plants, the fitness of individual fungi, and consequently community composition, varies along gradients of temperature, moisture and a suite of other environmental factors. Notably, however, when grown in isolation, many fungal taxa share overlapping optima toward the center of most environmental gradients (Barcenas-Moreno et al., 2009; Crowther and Bradford, 2013), and are forced to tolerate stressful conditions toward gradient edges (Figure 2). The relative importance of different abiotic factors can vary substantially between communities, but observational studies and multi-factor climate change experiments provide some consistent trends. Most fungi can tolerate a relatively large degree of variation in pH and temperature (Kardol et al., 2010), but are highly responsive to shifts in nutrient status and, in particular, water availability (e.g., Schimel et al., 2007; Kardol et al., 2010; Manzoni et al., 2014). Indeed, moisture stress (caused by disturbance, toxins, fire, drought or freezing) emerges as the dominant abiotic factor regulating the outcome of competitive fungal interactions (Magan and Lacey, 1984; Boddy, 2000). Because fungal cells are semi-permeable and in constant contact with their environment, the physiological challenge of maintaining water potential is essential to sustain turgor pressure, nutrient acquisition via substrate diffusion across membranes and to avoid desiccation (Schimel et al., 2007).

In the vast majority of terrestrial ecosystems, fungi experience drought stress to some extent, during either chronic or pulse events (Schimel et al., 2007). The accumulation of osmolytes reduces internal water potential and limits osmotic losses. Polyols including glycerol, erythritol, and mannitol are commonly regarded as the primary fungal osmolytes (Dijksterhuis and de Vries, 2006), and can constitute between 10 and 60% of total cytoplasmic constituents during stressful conditions (Schimel et al., 2007). Although the specific functions of different polyols vary among fungi (Solomon et al., 2007), their increased production under dry conditions suggests that they constitute a form of induced resistance to drought stress (Son et al., 2012). Heat-shock proteins are also induced to provide structural support, helping to maintain enzyme activity under stress. Chaperone molecules, for example, maintain enzyme-binding sites and facilitate conformational changes under dry conditions (Cowen, 2009). Water loss can also be physically restricted with the use of hydrophobic cell wall proteins, melanin and calcium oxalate crystals on mycelial surfaces (Unestam and Sun, 1995; Fernandez and Koide, 2013). An inability to express these physiological traits under stressful conditions generally forces fungi into dormancy, or ultimately cell death. Although most of these traits are associated with general mechanisms to tolerate a variety of other environmental stressors (e.g., heat, acidity; Figure 1), the relative trait expression (constant or potential) will provide a strong indication of a species' ability to persist at a given point along soil moisture gradients (Lennon et al., 2012).

Depending on whether stress-tolerance traits are constitutive or induced, cell maintenance can be energetically expensive, occurring at the cost of other metabolic processes. The cost of constitutive melanin production is, for example, relatively low, with growth rates of melanised individuals being almost equivalent to their non-melanised conspecifics (Fernandez and Koide, 2013). In contrast the induced synthesis of osmolytes and anti-shock proteins consistently leads to sub-optimal cell growth and sporulation (Dijksterhuis and de Vries, 2006). This trade-off between induced stress-tolerance and cell functioning is apparent at the genetic level. The environmental stress response (ESR), a common gene expression response to various biotic and abiotic stressors that has been conserved across the fungal kingdom, generally involves the expression of over 300 genes and the concurrent repression of ~600 others associated with cell maintenance and growth (Gasch, 2007).

Although the costs of induced stress-tolerance are apparent in most fungi, the degree to which cell growth is limited under sub-optimal conditions varies continuously among species; fungi adapted to specific stressors have the capacity to minimize the number of stress genes activated and repressed (Gasch, 2007). At least in some fungi, an epigenetic process governs this “adaptation.” In short, a genetic cascade initiates the expression of stress response genes, and then epigenetic regulation relies on changes in chromatin state and/or nuclear compartmentalization that silences all but a few necessary alleles (Verstrepen and Fink, 2009). This environmental selection and adaptation is likely to explain the niche differences observed between wet-adapted specialists and dry-adapted generalists (Lennon et al., 2012). The environmental gradient therefore acts as a trait filter, allowing inferences about species fundamental niches based on the relative expression of specific functional traits.

### Interaction milieu

Contracting fundamental into realized niche spaces requires consideration of biotic interactions, the nature of which are contingent upon the environmental conditions. Drought-tolerant fungi are not dry specialists, but “generalists,” with the potential to exist along a broad moisture-gradient (Lennon et al., 2012). Drought-intolerant fungi, in contrast, can be considered “specialists,” capable of competing under a narrow range of mesic conditions. Drought-tolerant and -intolerant fungi are therefore conceptually analogous to gymnosperm and angiosperm trees, respectively. For example, many coniferous trees have fundamental niche optima that fall in mesic temperate climate but are forced to tolerate colder and drier climates by competitively dominant angiosperms (Bond, 1989). We suggest that competitive ability similarly interacts with climate tolerances to shape the structure of fungal communities.

Under the CSR framework, ruderal and competitive species invest heavily in dispersal and competitive ability, respectively (Grime, 1977). Although the trade-off between these biotic processes undoubtedly exists in the fungal system (Kennedy et al., 2011), the high dispersal rates of most fungi, relative to those of plants and animals, is likely to promote the relative importance of competition, at least at local scales. Dispersal potential can favor early colonizers, with priority effects that determine community development (Fukami et al., 2010), but theoretical models predict that in communities experiencing high dispersal rates, survival in a given environment is primarily contingent upon the ability to compete (Mouquet and Loreau, 2003; Kneitel and Chase, 2004).

In most ecological systems, competition is largely determined by differing abilities to access limiting resources. This is apparent for certain plant symbionts (Chagnon et al., 2013), but most free-living fungal communities are governed by combat (Boddy, 1993, 2000; Saunders et al., 2010; Crowther et al., 2012a). That is, competitive interactions are determined by a species' ability to attack, or withstand attack, from antagonistic competitors. The importance of fungal combat as a structuring force in free-living fungal communities is apparent in that antagonistic ability comes at the cost of multiple other trait complexes. For example, despite the highly combative nature of *Resinicium bicolor* under optimal environmental conditions, its inability to remain combative during invertebrate grazing or temperature stress prevented the exclusion of fungal competitors in soil microcosms (Crowther et al., 2012b). Thus, the ability of a fungus to persist under a given set of abiotic conditions is immaterial if it cannot effectively overcome co-occurring combatants.

Various strategies allow fungi to attack opponents (e.g., gross mycelial contact, mycoparasitism, chemical interference; Boddy, 1993) and defend against attack (e.g., detoxification, structural alteration of toxins, exudation of absorbed toxins; Saunders et al., 2010). These interactions can be explored using pair-wise combinations of cultured fungi, either in a factorial design or by identifying the relative suppression of a common indicator species (Gaudet and Keddy, 1988). Pair-wise interactions are, however, often non-hierarchical and diffuse, so competitive ability based solely on pair-wise combinations can be misleading (Boddy, 2000). An alternative approach to rank competitors is to explore the growth of individuals within multi-species complexes, relative to growth in the absence of competitors (Fukami et al., 2010). Both of these approaches can generate combative trait complex hierarchies, providing a continuous scale with which to test for the dominant trade-offs that govern coexistence (Kneitel and Chase, 2004).

**Figure 3:**
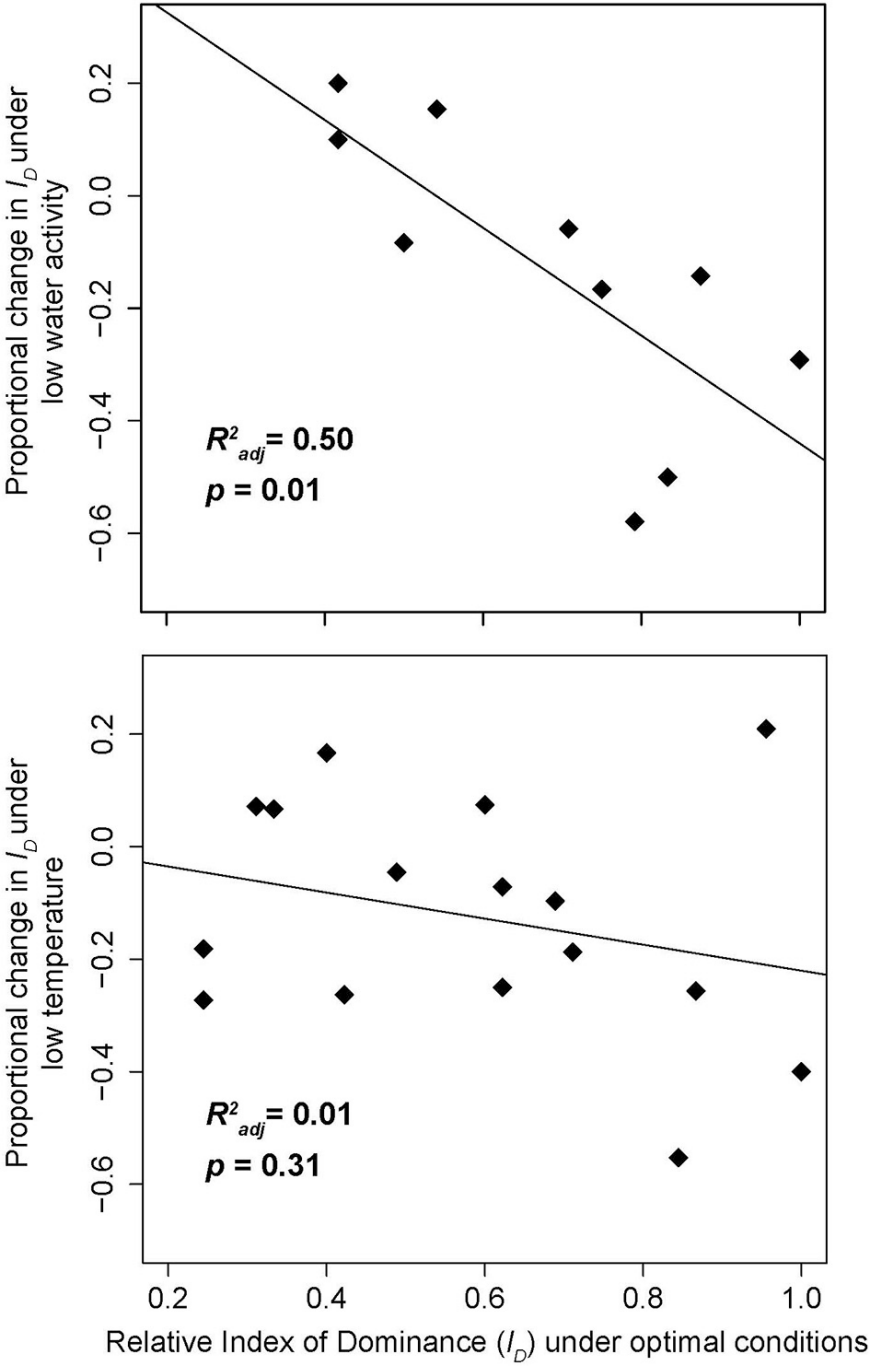
**The effect of reduced water activity (the partial vapor pressure of a substance) and decreased temperature on fungal combative ability**. Data are adapted from Tables 3,6 in Magan and Lacey (1984). Fungi that are the most combative under optimal conditions (25°C and water activity [*A_w_*] of 0.98) have a greater reduction in competitive ability under reduced water activity (*A_w_* = 0.90) than under reduced temperature conditions. Index of Dominance (*I_D_*) was assessed for each fungus by quantifying the outcomes of all pairwise competitions, with higher numbers indicating more competitive fungi (see Magan and Lacey, 1984 for details). (a) “Low water activity” refers to *A_w_* = 0.90, holding temperature constant at 25°C (6 of the 16 fungi had missing *I_D_* values at this water activity level); (b) “Low temperature” refers to 15°C, holding *A_w_* constant at 0.98. Relative *I_D_* values were calculated by dividing each fungus' *I_D_* by the maximum *I_D_* across all species. *R*^2*adj*^ and corresponding *p*-values were obtained by simple linear regression of relative *I_D_* vs. proportional change in *I_D_*.

The tendency of drought stress to alter these combative hierarchies (Boddy, 2000) provides strong evidence for a trade-off between drought stress and combative ability. Indeed, Magan and Lacey (1984) estimated an index of dominance (*I*_D_: a numerical score for combative ability) for multiple competing fungi in pairwise combinations in agar cultures, showing that both drought and temperature stress can alter the fungal dominance hierarchy. Re-analysis of this initial data reveals that the magnitude of the drought effect correlated strongly with initial combative ability (recorded under optimal conditions); the most antagonistic species experience the greatest competitive losses during drought stress. In contrast, whilst there was some evidence that temperature stress drives a similar relationship, the trade-off is substantially weaker, and the negative effects of heat stress are relatively consistent across all species (Figure 3; Magan and Lacey, 1984). Based on the strength and consistency of the relationship between drought-tolerance and combative ability (Magan and Lacey, 1984; Lennon et al., 2012), we hypothesize that this dominance-tolerance trade-off is likely to be a predominant mechanism structuring free-living fungal communities in predictable ways across time and space.

A mechanistic understanding of these dominance-tolerance trade-off in fungi requires the consideration of true traits associated with combat. The value of linking trait complex values to true traits is well recognized in other systems where, for example, plant height can provide a good approximation of the light competition milieu (McGill et al., 2006). However, linking true traits with fungal combative ability has proven challenging due, in part, to the variety of complex mechanisms of fungal antagonism (Hynes et al., 2007). Growing evidence suggests that fungal combat may be analogous to that observed in clonal animals (e.g., corals; Maguire and Porter, 1988), being governed by traits including growth rate (Boddy, 2000), antibiotic (Aguilar-Trigueros et al., 2014) and toxic secondary metabolite production (e.g., sesquiterpenes; Hynes et al., 2007; Saunders et al., 2010). We are unaware of studies that explore the relative importance of different combat or defense traits across a broad selection of fungi. Identifying a trait or a suite of traits that explains the fungal competition milieu, even within subsets of closely-related, or functionally similar fungi, will be an invaluable step in the formalization of trait-based approaches for understanding realized fungal niche spaces.

As with stress tolerance, fungal combative traits can be either induced or constitutive, but the energetic and genetic costs of combative trait expression require further exploration (Saunders et al., 2010). Initial evidence suggests that, in highly combative species, the genes associated with antagonism are clustered together on chromosomes (Mousa and Raizada, 2013), making these fungi highly susceptible to stress-induced gene silencing (Gasch, 2007). It is likely that dormancy represents an important mechanism for these fungi to avoid short-term periods of drought (Manzoni et al., 2014), but leaves them vulnerable to attack from stress-tolerant species in the longer term. In contrast, defensive traits of the less combative stress-tolerants are expected to be more constitutive, requiring minimal gene activation or deactivation during interactions (Iakovlev et al., 2004). This potential trade-off between induced attack and constitutive defense requires further exploration across a broad range of species, but potentially might explain shifts in fungal communities observed along moisture gradients. The highly “combative” species that we describe, therefore, reflect the “competitive” fungi described in Grime's CSR theory (Grime, 1977), although the fungi adapted to xeric conditions are simply those species capable of maintaining intermediate expression of combative traits under stressful conditions (see Box 1).

Box 1 **The dominance-tolerance trade-off model.**

We provide a mathematical framework to illustrate how trade-offs in fungal traits are likely to influence the community organization of species with overlapping environmental optima. Survival in a given environment is contingent on a fungus' ability to compete. This generally requires the diversion of energy from growth and maintenance toward the production of combative allelochemicals and altered growth strategies (Boddy, 2000). Fungal growth can therefore be modeled via a modified “competition for energy” model, as originally given in Schoener (1973). In Schoener's formulation, there is an indirect energetic cost of interaction between two species. We build on this concept by modeling the competition between fungi as having a direct energetic cost. The growth rates of two interacting species can thus be modeled by:

$$\begin{array}{l} \frac{dB_{1}}{dt}=\;r_{1g}(g)\cdot \left[E_{1}(g)-\;M_{1}(g)-C_{1}(g)\right]\cdot B_{1}-h_{1,2}\left(B_{1},B_{2},C_{1},C_{2},D_{1,2}\right)\\ \frac{dB_{2}}{dt}=\;r_{2}(g)\cdot \left[E_{2}\left(g\right)-\;M_{2}\left(g\right)-C_{2}(g)\right]\cdot B_{2}-h_{2,1}\left(B_{1},B_{2},C_{1},C_{2},D_{2,1}\right) \end{array}$$

Where *B_i_* is the biomass of fungus *i; g* is a given environmental condition along a gradient; *E_i_(g)* is net energy intake (after enzyme production and nutrient uptake) per unit biomass per unit time; *M_i_(g)* is the maintenance energy cost per unit biomass per unit time; *C_i_(g)* is energy invested in competition and combat per unit biomass per unit time; *r_i_(g)* is the energetic cost of building new cells, in terms of unit biomass per unit energy (i.e., growth rate per unit energy); and *h_i,j_*(·) is a function representing the death rate of fungus *i* as a function of the biomass of fungi *i* and *j*, the energy invested in competition by both *i* and *j*, and the “degree of interaction” (*D_i,j_*) between *i* and *j*. The functions *E_i_, M_i_, C_i_*, and *r_i_* all depend on the environmental condition, *g*.

**Figure 4:**
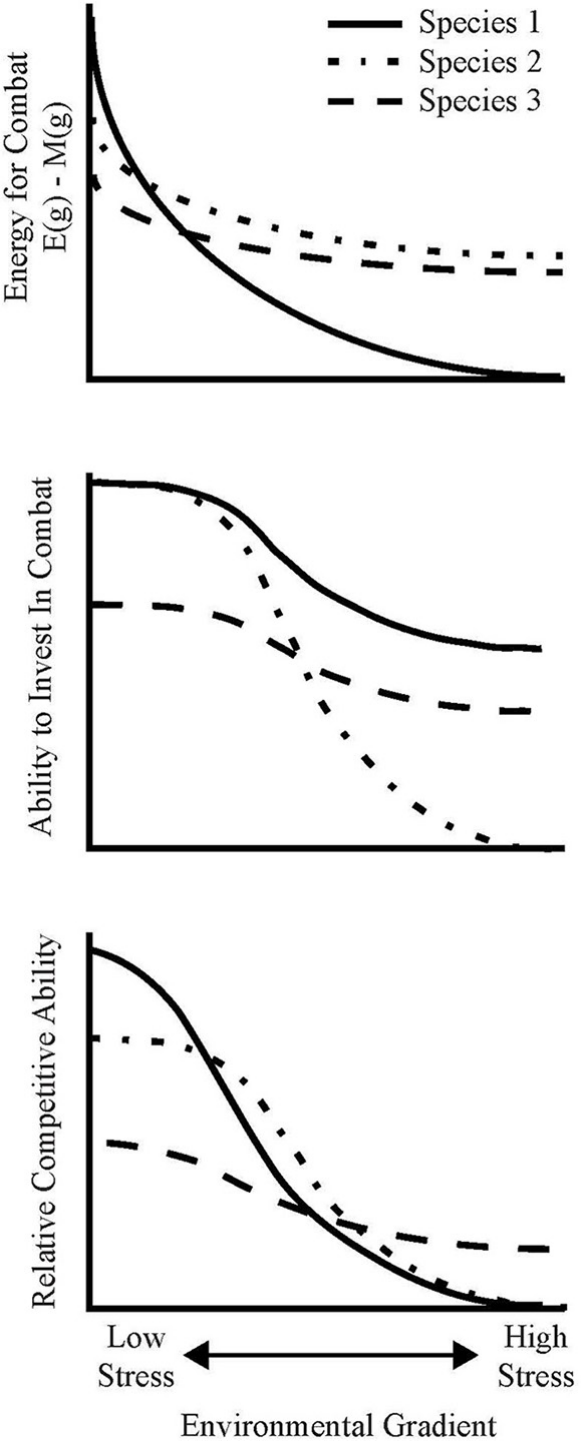
**Conceptual diagram to illustrate how differential investment of energy in stress-tolerance and combative ability can influence the community organization of species with overlapping fundamental niche spaces along environmental gradients**. The different lines refer to do individuals with different life-history strategies: species 1 invests heavily in combat but experiences substantial energetic limitation under stress, whilst species 2 experiences a genetic trade-off whereby combative ability is limited by the activation of environmental stress response genes, and species 3 is a specialist stress-tolerant species, capable of activating stress-response genes with minimal deactivation of combative genes (see Box 1).

Regardless of the specific forms of *E_i_, M_i_, C_i_, r_i_* and *h_i,j_*, it follows that, at a minimum, each fungus must ensure that *E*(*g*) − *M*(*g*) ≥ *C*(*g*) and *r*(*g*) ≥ 0 to survive in a given environment *g;* otherwise growth would be negative. These inequalities give rise to a set of intuitive trade-offs between stress tolerance and competitive ability:

- *Energetic limitation under stress*, where *E*(*g*) − *M*(*g*) decreases substantially along the gradient due to increased maintenance demands and/or decreased functional ability (either reduced enzyme production or affinity). Even if the fungus is able to invest most of the net remaining energy in combat, this restricted net energy intake places an upper bound on the amount of energy that it has to invest, thus limiting its competitive ability, growth or maintenance under stressful conditions (Figure 4, Species 1). If *r*(*g*) remains very large under stress, this species may persist at the landscape-level as a ruderal species, but its existence would be highly contingent on priority effects and on the biomass of its surrounding competitors.
- *Genetic limitation under stress*, where the fungus activates specific stress-response genes to ensure non-negative growth under stressful conditions, (i.e., to ensure that *r*(*g*)[*E*(*g*) − *M*(*g*)] ≥ 0). In doing so, the fungus necessarily deactivates genes that correspond to competitive ability. Thus, while *E* (*g*) − *M*(*g*) or *r*(*g*) need not substantially differ under high- and low-stress environments, the fungus is fundamentally unable to invest the leftover energy in competition under high-stress environments (Figure 4, Species 2).
- *Specialist stress tolerance*, where a fungus maintains competitive ability under high stress by avoiding substantial energetic, growth, and genetic limitations relative to stress-intolerant fungi. This could be achieved through an efficient epigenetic down-regulation of stress response genes or by the presence of constitutive stress-tolerance traits. For such fungi, it must be that both *E*(*g*) − *M*(*g*) and *r*(*g*) remain relatively large even under stressful conditions, and that the mechanisms by which it ensures positive net energy and growth do not substantially inhibit its ability to invest the net energy in antagonism (Figure 4, Species 3). The lack of a “Hutchinsonian demon” (a species capable of dominating in all environments) implies that the ability to avoid energetic, growth, and genetic limitations in stressful conditions must correspond to reduced relative competitive ability under optimal conditions, but the genetic basis for this trade-off remains unclear.

These three fungi represent examples selected from a continuous scale of life-history strategies, each of which may be employed to achieve the maximum possible combative ability under certain environmental condition. From this model it also follows that indicators of fungal survival (e.g., growth or respiration) in the absence of competition is not a meaningful surrogate for competitive ability, and is thus not useful for identifying the realized niche of a fungal species under the interaction milieu. Indeed, fungi with energetic and genetic limitations may exhibit growth and activity under stress when measured in isolation, but be out-competed by stress-tolerant species in the interaction milieu.

### Performance currency

A performance currency is a measurable characteristic that relates to the performance (or competitiveness) of individuals under a given environment. The traditional approaches for exploring fungal niche spaces involve measuring changes in biomass, growth or respiration under varying abiotic conditions (e.g., Barcenas-Moreno et al., 2009; Lennon et al., 2012; Crowther and Bradford, 2013). These approaches allow us to visualize fundamental niche spaces (the conditions under which an individual can exist), but are unable to address biotic interactions, or identify the conditions under which fungi can compete and survive. An alternative approach, incorporating biotic and abiotic processes, is to record the outcomes of combative interactions (measured as likelihood of winning or relative growth) under different abiotic conditions (Boddy, 2000; Crowther et al., 2012b). This approach has the potential to approximate the environmental conditions under which dominance can switch, but practical limitations in pairing multiple species in a fully factorial design across a full environmental gradient restrict our capacity to predict realized niches or extrapolate to other fungi. Instead, attempts to relate competitive ability to environmental conditions have generally involved only a handful of fungi and lead to the perhaps trivial conclusion that competitive interactions are context-dependent (e.g., Crowther et al., 2011).

To overcome these limitations and identify how fungal survivorship varies continuously across environments in the context of biotic interactions requires the identification of performance currencies that reflect the interaction milieu. “Combative ability” or associated traits (e.g., toxin production) could provide such a performance currency in many free-living fungal communities. Estimating how relative combative ability shifts along environmental gradients can help to predict, not only the conditions under which a fungus can survive, but under which it can compete for survival (Figure 2). Despite the paucity of studies testing trade-offs in free-living fungi, the prevalence of drought stress and combat within the fungal system, and the energetic trade-offs associated with both, provide a testable framework through which we can visualize the realized fungal niche (Box 1).

Dominance-tolerance trade-offs are apparent across the fungal kingdom (Boddy, 2000; Gasch, 2007), but the importance of these opposing processes will be context-dependent. Combat is, for example, less important than nutrient acquisition for arbuscular mycorrhizal fungi (Chagnon et al., 2013), and temperature stress is likely to exert a greater selection pressure than drought stress for the internal pathogens of mammals (Kraus and Heitman, 2003). Multiple trade-offs are likely to influence different components of the same community simultaneously. For example, a colonization-competition trade-off has been highlighted as a potential mechanism driving successional dynamics in ectomycorrhizal communities in woodland soil (Kennedy et al., 2011), whereas co-existing saprotrophic species are structured, in part, by trade-offs between combative ability and heat-tolerance (Crowther et al., 2012b). The limited number of individuals represented in these mechanistic studies, however, precludes robust examination and extrapolation of these trade-offs, and the restricted number of traits investigated limits our understanding of their relative importance within communities. Comparing the strength of different trade-offs, between and within communities, is essential to identify the dominant structuring forces within fungal communities and, therefore, the appropriate performance currencies necessary to untangle niche spaces.

## Linking traits to the environment

Once the dominant traits structuring communities are known they can be linked to the environmental conditions under which those individuals exist, to establish a mechanistic understanding of patterns of fungal biogeography and successional dynamics (Engelbrecht et al., 2007). The traits of individual, free-living fungi have yet to be linked explicitly to the environment, but the observed clustering of species within local habitats provides compelling evidence for the importance of habitat filtering at small spatial scales (e.g., Fukasawa et al., 2008; Feinstein and Blackwood, 2013; Crowther et al., 2014). This is especially apparent in the extremophile fungi (Kogej et al., 2007), whose efficient stress responses allow them to compete under temperature and moisture conditions far outside the ranges of most other species. Similar trends exist over time in patterns of fungal succession on decaying wood. For example, fungal diversity is greatest at mid-stages of wood decomposition (Rajala et al., 2011), where combat under optimal conditions is likely to be intense (Boddy, 2001). As decomposition progresses, changes in the chemical composition and physical structure of wood lead to extreme moisture conditions (either too moist or too dry), and/or decreased nutrient availability. These changing environmental conditions eventually select for fungi that can tolerate stressful conditions (Boddy, 2001) or for mycorrhizal fungi (Rajala et al., 2011), whose alternate nutrient-acquisition strategies (e.g., carbon provision by the host plant) provide a competitive advantage under energy-limitation.

Although the role of niche processes in structuring fungal communities is consistently supported at fine spatial scales, recent studies provide limited evidence for habitat filtering at regional and continent-scales (Martiny et al., 2006; Wu et al., 2013; Cline and Zak, 2014; Talbot et al., 2014). By focusing on taxonomic distributions, these studies highlight the importance of dispersal limitation and neutral processes in fungal taxonomic organization. However, we argue that, because environmental conditions have consistently been shown to select for individuals displaying similar characteristics in small-scale studies, the exploration of trait variation at broad scales is also likely to provide reveal more predictable patterns in fungal organization (Talbot et al., 2014). That fungal taxonomic distributions do not appear to reflect environmental conditions provides evidence for a discontinuity between taxonomic identity and trait values. Nevertheless, the observation of trait distributions requires consideration of the different coexistence mechanisms, the relative importance of which remain poorly understood in fungal communities.

### Coexistence mechanisms and trait dispersion

Apparent in the majority of observational studies, habitat filtering generally leads to the clustering (under-dispersion) of similar species by selecting for traits that allow individuals to survive in a particular environment. In contrast, “biotic sorting” (competition) can discourage the coexistence of species with similar trait values (Adler et al., 2013; Smith et al., 2013). By promoting niche differentiation, competition for resources generally leads to the over-dispersion of traits values compared to expectations from null models. Despite considerable and growing appreciation for these contrasting coexistence mechanisms in aboveground ecology (e.g., Adler et al., 2013; Smith et al., 2013; Herben and Goldberg, 2014), only one study to date has explored the relative importance of habitat filtering and niche partitioning in the fungal kingdom. Maherali and Klironomos (2012) showed that competition is the dominant structuring force in arbuscular mycorrhizal communities, preventing functionally similar traits from co-existing at fine spatial scales. Drawing inferences from plant communities, it is likely that the relative importance of these processes is scale-dependent, with the effects of habitat filtering being more apparent at the broadest of spatial scales (Smith et al., 2013). This scale effect is also likely to vary between components of the fungal community, with the effect of habitat filtering on endophytes and free-living saprotrophs being apparent at the scale of individual plants and ecosystems, respectively (Saunders et al., 2010).

**Figure 5:**
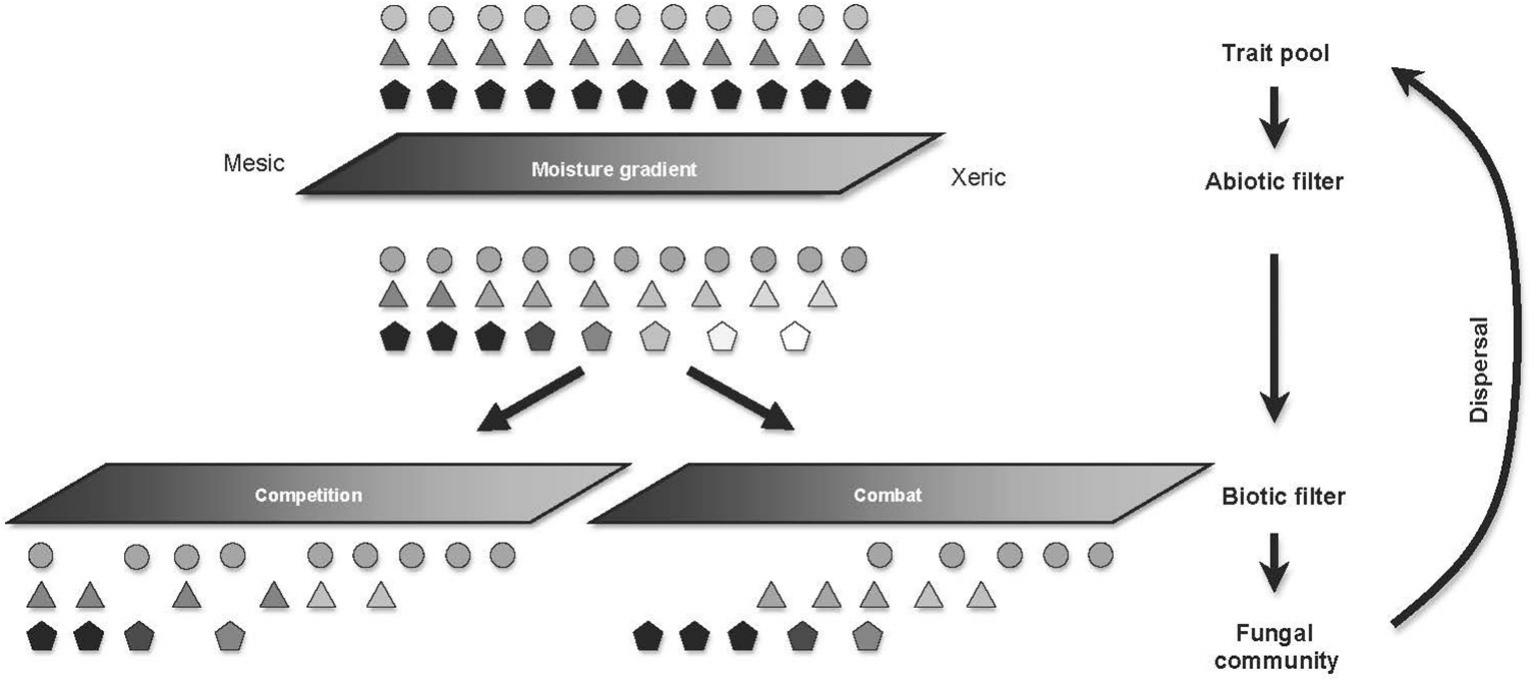
**Biotic and abiotic filters differentially influence trait organization along environmental gradients**. Similar shapes refer to individuals with similar stress-tolerance trait values, and the level of shading refers to relative combative ability of these individuals, with lighter individuals being less combative. On passage through the abiotic filter, stressful conditions reduce the survival and combative abilities of the most combative species, with negligible effects on the stress-tolerant species. Energetic costs of cell maintenance lead to reduced expression of combative traits, ultimately limiting the capacity of stress-intolerant species to compete under xeric conditions. However, the nature of the biotic filtering varies depending on the dominant interactive mechanism. Where combat (the direct killing of opponents) is the dominant interaction mechanism, the stress-intolerant individuals are capable of displacing the stress-tolerant individuals under mesic conditions, leading to under-dispersion of combative traits under mesic conditions. Conversely, competition for nutrients or refuges drives niche differentiation under optimal conditions, ultimately, resulting in trait over-dispersion in these optimal environments.

Along with spatial scale, the specific mechanisms through which organisms interact also require consideration when exploring how niche processes structure communities. Competition for resources, for example, generally promotes niche differentiation, but intense combat can select for the clustering of similar antagonistic or defensive traits (Mayfield and Levine, 2010; Herben and Goldberg, 2014). These opposing selection pressures can act simultaneously, leading to the over-dispersion of some traits and the under-dispersion of others within the same community (Figure 5; Herben and Goldberg, 2014). These dynamics could potentially contribute to the opposing patterns of community assembly observed between free-living, and mycorrhizal fungal communities in Australian sclerophyll forest (Beck et al., in preperation). Exploring trait dispersion across environments therefore requires consideration of the functional roles of those traits and the specific mechanisms through which they promote coexistence.

The use of fungal response traits is, therefore, not only essential to identify the dominant trade-offs that govern a species' potential distribution across environments, but also for understanding how coexistence mechanisms operate within communities. Exploring the equalizing and stabilizing mechanisms governing trait dispersion in the fungal kingdom is essential if we are to understand how niche processes affect patterns of diversity and community responses to environmental change (Adler et al., 2013). Until we have a grasp on these mechanisms, we will be limited in our capacity to explain or predict accurately community patterns observed in broad-scale observational studies. Being small, culturable and rapidly manipulated within laboratory environments, fungi are ideal model organisms to test these mechanisms and explore the predictions of current trait-based models of coexistence (Maherali and Klironomos, 2012).

## Conclusions

Their capacity for macromolecule degradation allows fungi to fill essential roles in the cycling of nutrients, detoxification of soil and regulation of both autotrophic and heterotrophic productivity (Hättenschwiler et al., 2005). Despite substantial variation between species, our understanding of fungal-mediated biogeochemical processes generally originates from species- or plot-level studies, limiting our capacity to extrapolate effects to regional and global scales. By linking physiological, morphological and biochemical properties of fungi with their environment, the trait-based approach that we outline can provide mechanistic insights into how fungal characteristics vary over time and space. The coupling of these response traits with effect traits can then facilitate the scaling of processes recorded in single sites/species to broader spatial-scales (Lavorel and Garnier, 2002; Pakeman, 2011; Koide et al., 2014). Where habitat filtering is a dominant coexistence mechanism governing trait distribution, relationships between environmental conditions and fungal effect traits are likely to be strong. Indeed, the trade-offs between abiotic stress tolerance and enzyme production are explicitly considered in recent decomposition models (Sinsabaugh et al., 2008; Allison, 2012); re-allocation of energy from enzyme synthesis to osmolyte production under increasing drought stress is a dominant mechanism governing differences in mineralization rates across landscapes (Manzoni et al., 2014). The relationships between response and effect traits are, however, rarely so direct (Pakeman, 2011), due, in part, to the multiple interacting coexistence mechanisms that can lead to complex trait mixtures. It is possible, for example, that the costs of combat under optimal moisture conditions (approximately −0.3 MPa; Lennon et al., 2012) are energetically equivalent to those involved with stress-tolerance under harsh conditions, with similar consequences for soil nutrient mineralization (Snajdr et al., 2011) and organic matter decomposition (Crowther et al., 2011) rates. Identifying the relative physiological costs of tolerating abiotic stress and biotic interactions (see Box 1) may be essential to improve the robustness of current decomposition models, and predict how the functioning of fungal communities varies between environments.

Despite the challenges involved in understanding communities governed by multiple coexistence mechanisms, community-level patterns of trait diversity can provide some insights into the functioning of fungal communities. The importance of fungal diversity for nutrient cycling (e.g., Fukami et al., 2010), host (including human) fitness (e.g., Ley et al., 2006) and ecosystem health (e.g., Hättenschwiler et al., 2005), is well appreciated, although the nature of fungal diversity-functioning relationships can vary substantially between communities (Nielsen et al., 2011). Biological diversity can influence ecosystem functioning, either by changing community-weighted mean trait values (related to the mass ratio hypothesis) or functional trait dissimilarity (related to non-additive community effects; Dias et al., 2013). The relative importance of these mechanisms remains unexplored in the fungal system, but may prove to be pivotal in predicting how changes in fungal diversity might affect ecosystem functioning across landscapes. Ultimately, we propose that the use of trait-based approaches is essential to explore whether the processes structuring fungal biogeography and functioning are unique, or equivalent to those in other taxonomic groups. The trait-based framework we provide highlights a number of key uncertainties that require testing in the fungal system, as they have the potential to advance the search for general principles in fungal ecology.

## Author contributions

TWC conceived the study. DSM designed the trade-off model and all authors contributed to manuscrpit writing and preperation.

### Conflict of interest statement

The authors declare that the research was conducted in the absence of any commercial or financial relationships that could be construed as a potential conflict of interest.
